# Quality of pilot trial abstracts in heart failure is suboptimal: a systematic survey

**DOI:** 10.1186/s40814-018-0302-8

**Published:** 2018-05-31

**Authors:** Godsent C. Isiguzo, Moleen Zunza, Maxwell Chirehwa, Bongani M. Mayosi, Lehana Thabane

**Affiliations:** 1 Department of Medicine, Groote Schuur Hospital and University of Cape Town, Cape Town, South Africa; 2 Cardiology Unit, Department of Medicine, Federal Teaching Hospital, Abakaliki, Nigeria; 3 Centre for Evidence-based Health Care, Division of Epidemiology and Biostatistics, Department of Global Health, Stellenbosch University, Cape Town, South Africa; 4 Department of Health Research Methods, Evidence, and Impact, McMaster University, Hamilton, ON Canada; 5 Biostatistics Unit/FSORC, Father Sean O’Sullivan Research Centre, St Joseph’s Healthcare-Hamilton, 50 Charlton Avenue East, 3rd Floor Martha Wing, Room H325, Hamilton, ON L8N 4A6 Canada

## Abstract

**Background:**

Pilot trials are miniature researches carried out with the sole aim of acting as the precursor for larger more definitive studies. Abstracts are used to summarize and introduce the findings to the reading audience. There is substantive empirical evidence showing that abstracts, despite their important roles, are not informative enough, lacking the necessary details. This systematic survey was designed to assess the quality of reporting of heart failure pilot trial abstracts. The quality of reporting was defined as the completeness of reporting based on adherence to the CONSORT extension for reporting of pilot trial abstracts. We also identified factors associated with reporting quality.

**Methods:**

We searched MEDLINE (PubMed), Cochrane Controlled Trials Register, Scopus, and African-wide information databases for abstracts from heart failure pilot trials in humans published from 1 January 1990 to 30 November 2016. These were assessed to determine the extent of adherence to CONSORT extension checklist for reporting of abstracts of pilot trials. We screened identified studies for inclusion based on title and abstract. Data were independently extracted by two reviewers using the checklist. We used regression analysis to assess the association between completeness of reporting (measured as the number of items in the CONSORT extension checklist for reporting of abstracts in pilot trials contained in each abstract) and factors influencing the quality of the reports.

**Results:**

Two hundred and twenty-eight (228) articles were retrieved, of which 92 met the inclusion criteria. The mean CONSORT extension score was 8.3/16 (standard deviation 1.7); the least reported items were the source of funding (1% [1/92]), trial registration (13% [12/92]), randomization sequence (13% [12/92]), number randomized to each arm (16% [15/92]), and number analyzed in each arm (16% [15/92]). Multivariable regression analysis showed that pharmacological intervention pilot trials [incidence rate ratio (IRR) = 0.88; 95% confidence interval (CI), 0.81–0.97] were significantly associated with better reporting. Other factors such as structured abstract (IRR = 1.10; 95% CI, 0.99–1.23) and CONSORT endorsement (IRR = 1.10; 95% CI, 0.99–1.23) only showed minimal relationship with better reporting quality.

**Conclusion:**

The quality of reporting of abstracts of heart failure pilot trials was suboptimal. Pharmacological intervention was significantly associated with better reporting. These findings are consistent with previous research on reporting of trials.

**Electronic supplementary material:**

The online version of this article (10.1186/s40814-018-0302-8) contains supplementary material, which is available to authorized users.

## Background

Numerous challenges confront readers while accessing the literature; these include but are not limited to the enormous volume of published work, the high cost of obtaining articles especially in resource-limited settings [1], and language constraints when articles are not written in users’ language [2]. The outcome of this has been an over-reliance on abstracts for articles as a one-stop point for most researchers. Abstracts of journal articles or scientific papers often provide readers with an overview of the content of the full article. As a result, researchers tend to rely on abstracts as a concise source of information [3] and in making decisions on which publications to read in detail [4]. Furthermore, researchers rely largely on the abstract when deciding whether to include an article in a systematic review [5]. All these factors make the abstract a key section of the scientific publication. It is, therefore, important that abstracts of articles are consistent with what is reported in the text and capture essential information.

Randomized control trials (RCT) constitute a significant portion of clinical studies and most times are the core component for systematic reviews. However, the quality of reporting of RCTs have over the years attracted many questions, mostly related to consistency and completeness of reports [6]. The Consolidated Standard for Reporting of Trials (CONSORT) checklist was conceptualized in 1996 to address these issues. Its publication and instant acceptance led to revisions in 2007 and 2010 [7, 8]. Many journals have adopted the CONSORT checklist, and it has been shown to improve the quality of reporting RCTs [9–11].

The overwhelming acceptance of CONSORT checklist has led to the development of extensions to incorporate other types of RCTs. Due to the need to widen the scope of the checklist, in 2016, the CONSORT extension checklist for abstracts of pilot trials was developed to aid adequate reporting of pilot trials [12], an important but often neglected arm of medical research [13].

We conducted this systematic survey to evaluate the quality of reporting of abstracts of pilot RCTs in heart failure published 1990–2016. Heart failure, defined as a clinical condition in which the heart does not pump blood sufficiently or does so at a higher pressure to maintain the body’s need [14, 15], has been a prominent cause of cardiovascular disease burden in Africa in the last two decades [16, 17]. It has also attracted many clinical trials in the last two decades (1990–2016), most of which were preceded by pilot trials [17]. The quality reporting is defined as complete reporting of the 16 items in the CONSORT extension checklist.

The aims of this survey are to (1) evaluate the quality of reporting of abstracts of pilot RCTs in the past 26 years (1990–2016), using the CONSORT extension for reporting of abstracts of pilot trials; (2) identify aspects of the checklist that are consistently reported; (3) identify factors associated with better reporting of abstracts; and (4) determine the quality (completeness) of abstracts of pilot trials.

## Methods

Randomized controlled pilot trials in heart failure published from 1 January 1990 to 30 November 2016 were searched for in line with the systematic survey method as previously described [17]. Abstracts were selected if they were described as random, randomly allocated, and randomized. We searched the MEDLINE (PubMed), Cochrane Controlled Trials Register, Scopus, and African-wide information databases (search strategy in the Additional file 1). We limited our search to pilot trial reports written in English language only. Two reviewers independently screened the identified papers and those finally selected to assess the quality of reporting of abstracts using the 16 items of CONSORT extension for reporting of abstract of pilot trials. We assigned a score of one to an item on the CONSORT checklist if the item was reported in the abstract. The overall quality of abstract was calculated as the proportion of “yes” responses. We classified abstracts that reported all the 16 items in the CONSORT checklist as adequate quality reporting.

We hypothesized that pilot trials published in high impact journals [18], published in CONSORT-endorsing journals [19, 20], those on pharmacological interventions [20], studies with large sample sizes [21], and industry-funded studies [22] would have better reporting quality.

The protocol for this systematic survey [17] was registered with PROSPERO (CRD42016049911) and written according to the Preferred Reporting Items for Systematic Reviews and Meta-Analysis Extension for Protocols (PRISMA-P) [23].

### Statistical analysis

The analysis was with IBM statistical package for social sciences (IBM SPSS) version 24 (IBM Corp., Armonk, NY) and STATA 9.0 (College Station, TX). We calculated the percentage of trials that scored yes on each of the 16 items and the associated 95% confidence interval (CI).

We reported categorical variables as count and percentages; continuous variables are summarized as mean (standard deviation (SD)) or median (interquartile range (IQR)). Incidence risk ratio (IRR) was calculated to identify factors associated with better reporting. Negative binomial regression was conducted to determine factors associated with better reporting quality.

## Results

**Fig. 1:**
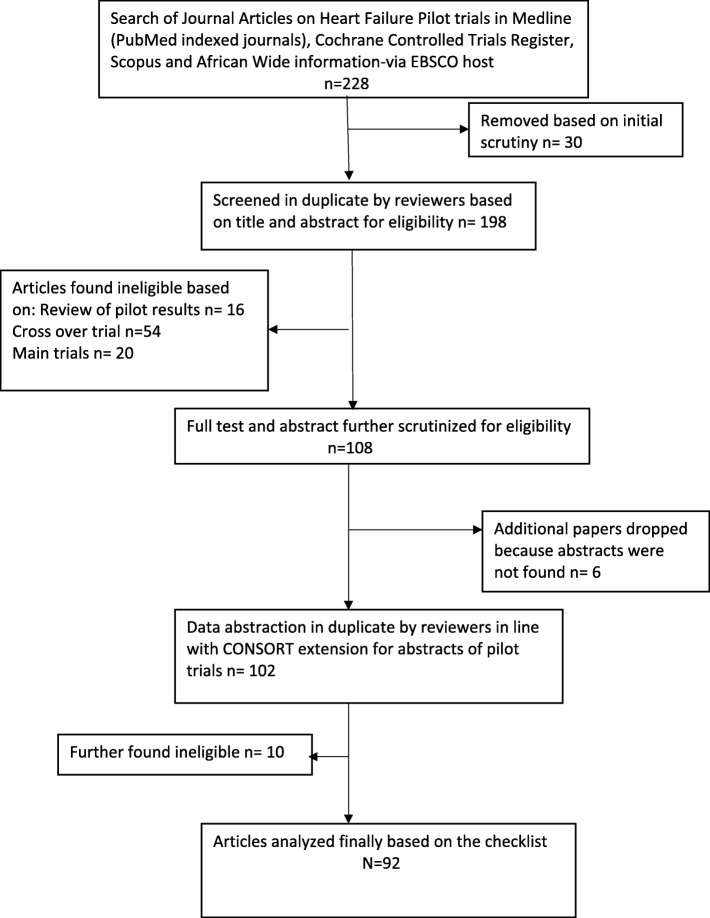
Study flow

Our search identified 228 articles; after the screening, one hundred and thirty-six articles were found to be ineligible based on several reasons (Fig. 1). A total of 92 articles from 48 journals were eligible; among these, 12 were conference presentations. The three highest contributing journals were *European Journal of Heart Failure* (9 articles; 9.8%), *American Heart Journal* (8 articles; (8.7%), *Journal of American College of Cardiology* (7 articles; 7.6%), and *Journal of Cardiac Failure* (7 articles; 7.6%).

### Study characteristics

The estimate of Kappa statistic for inter-rater agreement for screening publications for eligibility was 0.82 [95% confidence interval (CI), 0.76–0.87]. A majority (71%) of the studies were published between 2001 and 2016 (Table 1). Both pharmacological intervention studies and non-pharmacological intervention studies were 45 (49%), respectively; two studies (2%) had both pharmacological and non-pharmacological interventions. The abstract presentation was structured in 80% of the studies. In 62 of 92 (67%) of the studies, the CONSORT statement was not endorsed by the publisher. Most (74%) of the studies were conducted at a single site.

**Table 1:** Characteristics of included papers: *n* = 92

Characteristic	Count (%)/median (Q1; Q3)
Year of publication	
1990–2006	26 (28%)
2007–2016	66 (72%)
Intervention type (*n* = 90)	
Pharmacological	45 (49%)
Non-pharmacological	45 (49%)
Both pharmacological and non-pharmacological	2 (2%)
Abstract format	
• Structured	74/92 (80%)
• Unstructured	18/92 (20%)
CONSORT endorsement	
• Yes	30/92 (33%)
• No	62/92 (67%)
Number of sites	
• Single	61/82 (74%)
• Multiple	21/82 (26%)
Study duration in months (*n* = 68)	3.0 (2.6; 6.1)
Sample size (*n* = 88)	41 (24; 88)

*CONSORT* Consolidated Standards of Reporting Trials, *Q1* first quartile, *Q3* third quartile

### Quality of reporting of abstracts of pilot trials

None of the studies reported all the 16 items in the checklist (Table 2), the maximum reported number of items was 12, with a mean (SD) of 8.3 (1.7) items. The most reported item was the type of intervention intended for each group 99% (95% CI 91; 99), followed by specified objectives of the pilot trial 94% (95% CI 89; 99) and pre-specified outcome to address pilot trial objectives 97% (95% CI 90; 98).

**Table 2:** Publication adherence to CONSORT checklist for abstract of pilot trials *n* = 92

	Item	Criteria	Count	Percent (95% CI)
Title	Identifier	Title identifies the study is a randomized controlled pilot trial	65	71 (60; 79)
Description	Trial design	Description of pilot trial design (e.g., parallel or cluster)	30	33 (23; 44)
Method	Eligibility	Eligibility criteria for each participant	81	88 (78; 94)
	Settings	Setting where pilot was conducted	29	32 (22; 47)
	Interventions	Interventions intended for each group	91	99 (91; 99)
	Objectives	Specific objectives of the pilot trial	89	94 (89; 99)
	Outcomes	Pre-specified assessment or measurement to address the pilot trial objectives	89	97 (90; 98)
	Randomization sequence generation	Describe how participants were allocated to the interventions	12	13 (8; 19)
	Blinding (masking)	Whether or not participants, caregivers, and those assessing the outcomes were blinded to group assignment	20	22 (15; 31)
Results	Number randomized	Number of participants screened	15	16 (9; 26)
		Number randomized to each group for the pilot objectives	41	45 (36; 53)
	Recruitment	Trial status (for conference abstracts)	2	2 (5; 8)
	Numbers analyzed	Number of participants analyzed in each group of pilot objectives	15	16 (11; 24)
	Harm	Important adverse events or side effects	28	30 (23; 39)
Conclusion	Result interpretation	General interpretation of results of pilot trial	80	87(76; 93)
	Plans	Any implication for future trial	20	22 (15; 30)
Registration	Trial registration	Registration number of trial	12	13 (7; 21)
	Funding	Source of funding	1	1 (0.16; 6.9)

*CI* confidence interval

The least reported item was funding source 1% (95% CI 0.16; 6.9); however, 21 of 92 (22.8%) studies reported funding source in the main manuscript but not in the abstract. Inadequately reported items include the randomization method used 13% (95% CI 8; 19), trial registration information 13% (95% CI 7; 21), and the number of participants screened in each arm 16% (95% CI 9; 26). Recruitment status, an item in the CONSORT checklist that addresses conference presentation, was low with 2% (95% CI 5; 8) among the 12 conference abstracts.

Multivariable analysis (Table 3) of factors associated with reporting quality showed pharmacological intervention is significantly associated with better reporting quality (IRR 0.88; 95% CI 0.81; 0.97; *p* value 0.01), while structured abstract (IRR 1.10; 95% CI 0.99; 1.23; *p* value 0.05) did not have a strong association. Journals which endorsed CONSORT was not a significant factor (IRR 1.10; 95% CI 0.99; 1.23; *p* value 0.06).

**Table 3:** Univariate and multivariable analysis of factors associated with reported items

Item	Univariate		Multivariable	
	IRR (95% CI)	*p* value	IRR (95% CI)	*p* value
Year of publication				
• 1990–2006 • 2007–2016	0.99 (0.91; 1.08)	0.86	1.07 (0.91; 1.13)	0.74
Intervention type				
• Pharmacological • Non-pharmacological	0.93 (0.86; 1.01);	0.07	0.88 (0.81; 0.97)	0.01
Abstract format				
• Structured • Unstructured	1.11 (0.99; 1.24)	0.08	1.10 (0.99; 1.23)	0.05
CONSORT endorsement				
• Yes • No	1.07 (0.98; 1.16)	0.12	1.10 (0.99; 1.23)	0.06
Number of sites				
• Single • Multiple	1.08 (0.97; 1.22)	0.14	1.02 (0.92; 1.15)	0.62
Study duration*	1.03 (1.01; 1.06)	0.02		
Sample size*	1.01 (0.96; 1.07)	0.63		

*IRR* incidence rate ratio, *CI* confidence interval

*Incident rate for change in 1 unit on the log scale

## Discussion and conclusions

The CONSORT extension for reporting of abstracts of pilot trials was introduced in 2016 to standardize the reporting of abstracts of such studies. We undertook this survey to gauge the current practice using heart failure pilot trials as a sample to track reporting quality improvement expected with introduction and adherence to the extension. We found inadequate reporting quality in keeping with previous articles evaluating adherence to CONSORT checklist on abstracts [8, 24, 25].

The least reported item was the funding source, similar to findings in a previous study [26]. The full declaration of funding source in a publication can give the reader the opportunity to make their own assessment regarding potential conflict of interest. However, we noticed that 22/96 of the articles had information on funding in other places rather than the abstract, and this may not be unrelated to word count stipulation by individual journals. Often, this poses a challenge to authors on what to include in abstracts, and there may be the need for CONSORT extension checklist developers to consider this limitation.

The randomization sequence was also poorly reported. It is an item that can provide acceptable comparability between groups if properly reported. Some previous studies have commented on this methodological flaw [10, 27–29], explaining that this could be because of attachment of more relevance to clinical than the methodological aspect of RCTs. There has been corroborating evidence in other studies supporting a correlation between deficient reporting and poor trial methodology [30–32].

Many of the articles were also silent on the blinding, and when used, some failed to state the group blinded in the studies. Not being explicit about blinding erodes the integrity and internal validity of the reports, and it is a potential source of bias among the readers [33, 34]. Reporting on harm was low at 28%; this is an essential item as it informs the design and applicability of intended larger trials. Previously, this safety reporting has often been inadequate or neglected [35]. And, in literature, there is highly variable adherence to reporting of harm [36, 37].

Methodological aspects reported include intervention type, specific objective, and outcomes to be assessed. Many of the articles identified them as pilot trials; this was despite the constraint of word limit by journals, a reason often adduced as responsible for not including this [10, 26, 29]. Also, pilot trials with pharmacological intervention, those with structured abstracts, and those published in the journal that endorses CONSORT were more likely to report on the items on the checklist. The last point brings to fore why it is important for journals to have stipulated reporting format to ensure that the quality of abstracts’ report is improved.

Our study is limited in scope by using only articles published in the English language. We also used a recently produced checklist to evaluate publications done by authors who at the time of writing of the articles probably had no reporting checklist to follow.

In conclusion, the reporting quality of abstracts of heart failure trial measured by the number of items reported was suboptimal; the need to guard against this was the reason that informed the introduction of CONSORT extension checklist. The desire is that increasingly journals will demand adherence to the checklist by authors. Ultimately, we hope that there will be a marked improvement in the quality of report of abstracts in the coming years.

## Additional file

Additional file 1: Database search strategies. (DOCX 21 kb)

## References

[1] Godlee F, Horton R, Smith R (2000). Global information flow. BMJ.

[2] Scherer RW, Langenberg P, von Elm E (2007). Full publication of results initially presented in abstracts. Cochrane Database Syst Rev.

[3] The PME (2006). The impact of open access upon public health. PLoS Med.

[4] Barry HC, Ebell MH, Shaughnessy AF, Slawson DC, Nietzke F (2001). Family physicians’ use of medical abstracts to guide decision making: style or substance?. J Am Board Fam Pract.

[5] Smith H, Bukirwa H, Mukasa O, Snell P, Adeh-Nsoh S, Mbuyita S (2007). Access to electronic health knowledge in five countries in Africa: a descriptive study. BMC Health Serv Res.

[6] Berwanger O, Ribeiro RA, Finkelsztejn A, Watanabe M, Suzumura EA, Duncan BB (2009). The quality of reporting of trial abstracts is suboptimal: survey of major general medical journals. J Clin Epidemiol.

[7] Moher D, Jones A, Lepage L (2001). Use of the CONSORT statement and quality of reports of randomized trials: a comparative before-and-after evaluation. JAMA.

[8] Ghimire S, Kyung E, Kang W, Kim E (2012). Assessment of adherence to the CONSORT statement for quality of reports on randomized controlled trial abstracts from four high-impact general medical journals. Trials.

[9] Mbuagbaw L, Thabane M, Vanniyasingam T, Borg Debono V, Kosa S, Zhang S (2014). Improvement in the quality of abstracts in major clinical journals since CONSORT extension for abstracts: a systematic review. Contemp Clin Trials.

[10] Camm CF, Chen Y, Sunderland N, Nagendran M, Maruthappu M, Camm AJ (2013). An assessment of the reporting quality of randomised controlled trials relating to anti-arrhythmic agents (2002-2011). Int J Cardiol.

[11] Plint AC, Moher D, Morrison A, Schulz K, Altman DG, Hill C (2006). Does the CONSORT checklist improve the quality of reports of randomised controlled trials? A systematic review. Med J Aust.

[12] Eldridge SCM, Thabane L, Bond C, Hopewell S, Lancaster G. CONSORT extension guidelines for reporting pilot and feasibility. Trials. 2016;2(1):64 10.1186/s40814-016-0065-z PMC5153862 27965844

[13] Lancaster GA, Dodd S, Williamson PR (2004). Design and analysis of pilot studies: recommendations for good practice. J Eval Clin Pract.

[14] Yancy CW, Jessup M, Bozkurt B, Butler J, Casey DE, Drazner MH (2013). 2013 ACCF/AHA guideline for the management of heart failure: a report of the American College of Cardiology Foundation/American Heart Association Task Force on practice guidelines. J Am Coll Cardiol.

[15] Mayo Clinic. Heart Failure 2017 [cited 2017 15 June]. Available from: https://www.mayoclinic.org/diseases-conditions/heart-failure/symptoms-causes/syc-20373142.

[16] Hall MJ, DeFrances CJ, Williams SN, Golosinskiy A, Schwartzman A (2010). National hospital discharge survey: 2007 summary. Natl Health Stat Report.

[17] Isiguzo G, Zunza M, Chirehwa M, Mayosi BM, Thabane L (2017). Quality of abstracts of pilot trials in heart failure: a protocol for a systematic survey. Contemp Clin Trials Commun.

[18] Turner L, Shamseer L, Altman DG, Weeks L, Peters J, Kober T (2012). Consolidated standards of reporting trials (CONSORT) and the completeness of reporting of randomised controlled trials (RCTs) published in medical journals. Cochrane Database Syst Rev.

[19] Thabane L, Chu R, Cuddy K, Douketis J (2007). What is the quality of reporting in weight loss intervention studies? A systematic review of randomized controlled trials. Int J Obes.

[20] Samaan Z, Mbuagbaw L, Kosa D, Borg Debono V, Dillenburg R, Zhang S (2013). A systematic scoping review of adherence to reporting guidelines in health care literature. J Multidiscip Healthc.

[21] Borg Debono V, Zhang S, Ye C, Paul J, Arya A, Hurlburt L (2012). The quality of reporting of RCTs used within a postoperative pain management meta-analysis, using the CONSORT statement. BMC Anesthesiology.

[22] Lai R, Chu R, Fraumeni M, Thabane L (2006). Quality of randomized controlled trials reporting in the primary treatment of brain tumors. J Clin Oncol.

[23] Moher D, Liberati A, Tetzlaff J, Altman DG, The PG. Preferred reporting items for systematic reviews and meta-analyses: the PRISMA statement. PLoS Med 2009;6(7):e1000097. 10.1371/journal.pmed.1000097 PMC2707599 19621072

[24] Bigna JJ, Noubiap JJ, Asangbeh SL, Um LN, Sime PS, Temfack E (2016). Abstracts reporting of HIV/AIDS randomized controlled trials in general medicine and infectious diseases journals: completeness to date and improvement in the quality since CONSORT extension for abstracts. BMC Med Res Methodol.

[25] Ntala C, Birmpili P, Worth A, Anderson NH, Sheikh A (2013). The quality of reporting of randomised controlled trials in asthma: systematic review protocol. Primary Care Respir J.

[26] Hopewell S, Dutton S, Yu LM, Chan AW, Altman DG (2010). The quality of reports of randomised trials in 2000 and 2006: comparative study of articles indexed in PubMed. BMJ.

[27] Rios LP, Odueyungbo A, Moitri MO, Rahman MO, Thabane L (2008). Quality of reporting of randomized controlled trials in general endocrinology literature. J Clin Endocrinol Metab.

[28] Anttila H, Malmivaara A, Kunz R, Autti-Ramo I, Makela M (2006). Quality of reporting of randomized, controlled trials in cerebral palsy. Pediatrics.

[29] Nojomi M, Ramezani M, Ghafari-Anvar A (2013). Quality of reports on randomized controlled trials published in Iranian journals: application of the new version of consolidated standards of reporting trials (CONSORT). Arch Iran Med.

[30] Huwiler-Muntener K, Juni P, Junker C, Egger M (2002). Quality of reporting of randomized trials as a measure of methodologic quality. JAMA.

[31] Juni P, Altman DG, Egger M (2001). Systematic reviews in health care: assessing the quality of controlled clinical trials. BMJ.

[32] Montori VM, Wang YG, Alonso-Coello P, Bhagra S (2006). Systematic evaluation of the quality of randomized controlled trials in diabetes. Diabetes Care.

[33] Dickinson K, Bunn F, Wentz R, Edwards P, Roberts I (2000). Size and quality of randomised controlled trials in head injury: review of published studies. BMJ.

[34] Thornley B, Adams C (1998). Content and quality of 2000 controlled trials in schizophrenia over 50 years. BMJ.

[35] Ioannidis JP, Lau J (2001). Completeness of safety reporting in randomized trials: an evaluation of 7 medical areas. JAMA.

[36] Agha R, Cooper D, Muir G (2007). The reporting quality of randomised controlled trials in surgery: a systematic review. Int J Surg.

[37] Sinha S, Sinha S, Ashby E, Jayaram R, Grocott MP (2009). Quality of reporting in randomized trials published in high-quality surgical journals. J Am Coll Surg.

