# Supplementary material for: Quality of pilot trial abstracts in heart failure is suboptimal: a systematic survey
Source: Pilot Feasibility Stud. 2018 May 31;4:107. doi: 10.1186/s40814-018-0302-8 (PMC5977467; doi:10.1186/s40814-018-0302-8)
Supplement: Supplementary file 1 — Database search strategies. (DOCX 21 kb) [file 40814_2018_302_MOESM1_ESM.docx]

**Additional file 1: Database search strategies**

Medline

(((((Heart failure[MeSH Terms]) OR ((Heart failure OR paroxysmal dyspnea OR diastolic heart failure OR systolic heart failure OR Cardiac Failure OR Heart Decompensation OR Myocardial Failure OR Congestive Heart Failure OR Ventricular dysfunction OR cardiac insufficiency OR myocardial failure OR myocardial insufficiency)))) AND ((randomized controlled trial [pt] OR controlled clinical trial [pt] OR randomized controlled trials [mh] OR random allocation [mh] OR double-blind method [mh] OR single-blind method [mh] OR clinical trial [pt] OR clinical trials [mh] OR ("clinical trial" [tw]) OR ((singl* [tw] OR doubl* [tw] OR trebl* [tw] OR tripl* [tw]) AND (mask* [tw] OR blind* [tw])) OR (placebos [mh] OR placebo* [tw] OR random* [tw] OR research design [mh:noexp] OR (comparative study) OR (comparative studies) OR (evaluation studies) OR (evaluation study) OR follow-up studies [mh] OR prospective studies [mh] OR controlled [tw]OR controls [tw]OR control [tw] OR prospectiv* [tw] OR volunteer* [tw]) NOT (animals [mh] NOT human [mh]))))) AND (((Pilot projects[MeSH Terms]) OR Feasibility Studies[MeSH Terms]) OR ((pilot OR feasibility)))

Africa-Wide (Ebsco) and Web of Science

| Heart failure OR paroxysmal dyspnea OR diastolic heart failure OR systolic heart failure OR Cardiac Failure OR Heart Decompensation OR Myocardial Failure OR Congestive Heart Failure OR Ventricular dysfunction OR cardiac insufficiency OR myocardial failure OR myocardial insufficiency  AND  clinical trial OR randomized controlled trial OR randomised controlled trial OR random allocation OR double-blind OR single-blind OR placebo OR random research OR comparative study OR evaluation study OR follow up OR follow-up OR prospective OR control OR volunteer OR single mask OR double mask OR treble mask OR triple mask OR single blind OR double blind OR treble blind OR triple blind  AND  pilot OR feasibility  Filter by years (1 January 1990- 1 May 2016) |
| --- |

Scopus

| Heart failure OR paroxysmal dyspnea OR diastolic heart failure OR systolic heart failure OR Cardiac Failure OR Heart Decompensation OR Myocardial Failure OR Congestive Heart Failure OR Ventricular dysfunction OR cardiac insufficiency OR myocardial failure OR  myocardial insufficiency  AND  clinical trial OR randomized controlled trial OR randomised controlled trial OR random allocation OR double-blind OR single-blind OR placebo OR random research OR comparative study OR evaluation study OR follow up OR follow-up OR prospective OR  control* OR volunteer OR single mask OR double mask OR treble mask OR triple mask OR  single blind OR double blind OR treble blind ORtriple blind AND pilot OR feasibility  Filter by years (1 January 1990- 1 May 2016) |
| --- |
